# How does the onset of incontinence affect satisfaction with life among older women and men? Findings from a nationally representative longitudinal study (German Ageing Survey)

**DOI:** 10.1186/s12955-020-1274-y

**Published:** 2020-01-28

**Authors:** Elżbieta Buczak-Stec, Hans-Helmut König, André Hajek

**Affiliations:** Department of Health Economics and Health Services Research, Hamburg Center for Health Economics, University Medical Center Hamburg-Eppendorf, Martinistr. 52, 20246 Hamburg, Germany

**Keywords:** Cognitive well-being, Health-related quality of life, Incontinence, Life satisfaction, Longitudinal studies, Sex differences, Subjective well-being

## Abstract

**Background:**

There is a large body of evidence showing that incontinence is associated with decreased *health-related quality of life* (HRQoL). Moreover, while a few *cross-sectional studies* have shown that incontinence is associated with decreased *life satisfaction*, there is a lack of studies regarding whether the onset of incontinence influences life satisfaction of affected individuals *longitudinally*. Thus, the objective of this study is: (i) to investigate the impact of incontinence on life satisfaction and (ii) whether this effect differed between women and men using a large population-based sample longitudinally.

**Methods:**

Longitudinal data from 2008 to 2014 were retrieved from a nationally representative sample (9869 observations in regression analysis) of community-dwelling individuals aged 40 years and over (German Ageing Survey, DEAS). Physician-diagnosed incontinence was reported by respondents. Life satisfaction was quantified using the well-established Satisfaction with Life Scale. Linear fixed-effects regressions were used.

**Results:**

After adjusting for potential confounders (e.g., self-rated health or depression), regressions revealed that the onset of incontinence was associated with a decline in life satisfaction in men (β = −.25, *p* < .01), but not in the total sample and in women. These differences were significant (*p* < .05). In a further sensitivity analysis, individuals with cancer were excluded. However, in terms of significance and effect size, the impact of incontinence on life satisfaction in men remained almost the same in both models.

**Conclusions:**

The onset of incontinence markedly reduces life satisfaction among men aged 40 and over. Interventional strategies to postpone incontinence may assist in maintaining life satisfaction in men.

## Background

Urinary Incontinence (UI), according to the International Continence Society (ICS), is a complaint of any involuntary leakage of urine [1]. Fecal incontinence (FI) refers to accidental leakage of liquid or solid stool [2]. Both UI and FI symptoms are much more common in older age. While UI affects up to 63% of women aged 80+ and up to 23% older men [3], around 6.0% of women and 1.9% of men reported both UI and FI [4].

While a weak pelvic floor may contribute to incontinence among women, incontinence among men is mostly caused by various, more complex reasons including treatment of prostate cancer, neurological diseases, cognitive changes or loss of mobility [5]. Both symptoms are associated with numerous adverse health factors including depression, hypertension, diabetes mellitus, stroke, obesity, functional impairment and cognitive impairment [3, 6]. Due to demographic change in the coming decades, incontinence is an increasing health concern.

There is a large body of evidence showing that incontinence is associated with decreased *health-related quality of life* (HRQoL) [7, 8]. Moreover, while a few *cross-sectional studies* have shown that incontinence is associated with decreased *life satisfaction* [9–11], there is a lack of studies regarding whether the onset of incontinence influences life satisfaction of affected individuals *longitudinally*.

Based on cross-sectional data, some researchers have reported differences in life satisfaction when comparing group of individuals experiencing incontinence with continent individuals [10, 11]. Moreover, Dugan et al. in the research based on 425 incontinent adults (average age 72.5) drew attention to changes in (generic) life satisfaction. He concluded that life satisfaction is affected, among other things, by the amount of urine loss [12]. A few authors have assessed satisfaction with life and incontinence resulting from various medical conditions. In a study based on 50 patients after transurethral prostatectomy (average age 64.5 years), improved symptoms of incontinence, which resulted from pelvic floor muscle exercises, lead to greater satisfaction with life [13]. Edwards et al. [14] in the study of 361 community-dwelling individuals after stroke onset, showed that urinary incontinence was associated with poor life satisfaction. Apart from incontinence, previous studies showed that life satisfaction among people in their second half of life can be influenced by set of other factors. It has been suggested that, among others, physical health, depression, onset of health symptoms, age and socioeconomic status could influence satisfaction with life [10, 15, 16].

Although HRQoL and life satisfaction are similar constructs, there is an essential difference between them. HRQoL reflects mainly (self-perceived) individuals’ health. HRQoL includes factors that both pertain to an individual’s health and that impact upon an individual’s life [17, 18].

In contrast, life satisfaction refers to the individual’s assessment and/or judgement of their well-being against their own criteria and standards [19]. As per Diener [20, 21], life satisfaction is a component of subjective well-being. Life satisfaction includes the global assessment of an individual’s life [21]. The well-established measure of life satisfaction - Satisfaction with Life Scale (SWLS) [19] – is a global assessment of one’s perceptions of and attitudes towards one’s life, and does not center around any specific domain e.g. health. The importance of specific aspects or domains of life is not predetermined by the measure. That is, each individual decides which domain of life is important for them, according to their own values, and weights this in their overall assessment of life accordingly.

Life satisfaction among citizens is key goal among nations (e.g. gross national happiness) which could create beneficial societal outcomes such as better health status. Therefore it is important to study the factors that influence life satisfaction [20, 22]. Furthermore, it has been demonstrated that affected individuals are wary of successful treatment possibilities for incontinence [3, 23]. Therefore, knowledge about the impact of incontinence on satisfaction with life may help to overcome skepticism around treatment, as treatments may assist in improving life satisfaction.

We hypothesize that the onset of incontinence decreases life satisfaction in the total sample as it may be perceived as a critical event. However, we hypothesize that the consequences of the onset of incontinence differs in women and men. Since incontinence is a common condition (in women), many women may treat it as a normal part of aging, and/or as a non-critical condition [3, 23]. The number of men suffering from incontinence is markedly lower. Thus, we assume that the onset of incontinence is a critical life event among men. Consequently, they may compare themselves with other men their age group not suffering from incontinence, and these negative health comparisons may lead to a decrease in life satisfaction [24]. Moreover, men suffering from incontinence may feel embarrassed, stigmatized or fear the reaction of others [25].

Therefore, the aim of this study was (i) to longitudinally investigate the impact of incontinence on life satisfaction and (ii) whether this effect differed between women and men using a large population-based sample. Potential gender differences were examined in this study because it has been shown that men and women differ in their predictors of life satisfaction [26].

## Methods

### Sample

Data were collected as part of the German Ageing Survey (DEAS), which was launched by the German Federal Government in the mid-1990s. It is a large nationwide, longitudinal study of the community-dwelling middle aged and older population in Germany (40 years and over). One of the main aims of this study is to investigate the process of ageing (e.g., health outcomes, or subjective well-being). Due to the cohort-sequential design it is possible to analyze intra-individual change [27].

Since 1996, every 6 years, new nationally representative cross-sectional baseline samples are drawn (*n* = 4838 in 1996; *n* = 3670 in 2002; *n* = 6205 in 2008; *n* = 6002 in 2014). In addition, longitudinal data for each baseline sample are collected (that is in 2002, 2008, 2011 and 2014). Since the third wave in 2008, the period between waves has been reduced to 3 years. Overall, the response rate was reasonably low in the DEAS study, however it is comparable to other German surveys [28]. The response rates for baseline samples decreased from 50.3% in 1996 to 27.1% in 2014 [27]. The retention rate (valid re-interviews) increased to 41.4% in 2014, based on the 2008 baseline. The sampling methodology and the cohort profile is described in further detail elsewhere [27].

Since the scope of our analysis was to investigate the longitudinal association between incontinence and life satisfaction, our regression analysis was restricted to the third (2008), fourth (2011) and fifth (2014) wave due to data restrictions (*n* = 9869 observations in fixed effects regression analysis). A question concerning incontinence was only included as part of the questionnaire in these waves.

### Dependent variables

The life satisfaction of individuals was quantified using the well-established Satisfaction with Life Scale (SWLS) [19]. The scale consists of five questions e.g. “In most ways my life is close to my ideal” or “If I could live my life over, I would change almost nothing”, each question is rated on 5-points scale from 1 - “strongly agree” to 5 - “strongly disagree”. The final SWLS score ranges from one to five where high values indicate high life satisfaction [29]. The scale demonstrates favorable psychometric properties [19].

### Independent variables

In the DEAS survey, respondents were asked to identify from a list of several illnesses, which illnesses they had been formally diagnosed with by their doctor. Incontinence was included in this list of illnesses. Participants received the list of the illnesses listed with the corresponding letters (A-T) (Appendix). The occurrence of incontinence was determined using responses to this section of the survey (self-reported from participants). The illnesses included in this list were selected based on, among other things, the Charlson Comorbidity Index [30] and consultations with specialists with a background in geriatrics.

With respect to control variables, we included age, gender, marital status (married, living together with spouse; other (divorced; widowed; single)) and labor force participation (working; retired; other: not employed). Furthermore, we controlled for the size of social network (defined as the number of the most important persons with whom individuals are staying in a regular contact with; range from 0 to 9), physical functioning, depression and self-rated health. Physical functioning was measured using the subscale “Physical Functioning” of SF-36 Short Form Health Survey (0–100 range) [31]. High values indicate good physical functioning. Depression was assessed using the Center for Epidemiological Studies Depression Scale (CES-D) [32], ranging from 0 to 45, with high values representing high depressive symptoms with the cut-off at ≥18. Self-rated health is a powerful indicator of clinical outcomes [33], with ratings of overall health ranging from very good (=1) to very poor (=5).

Based on theoretical considerations and empirical studies [9–14], these variables were selected. Thus, it was adjusted for age, marital status, employment status, number of important people in regular contact, self-rated health, physical functioning and depression in the main model. Stepwise regression models were not used.

In a sensitivity analysis, we extended our model to include level of education: low (0–2), medium (3–4), and high (5–6), according to the International Standard Classification of Education (ISCED-97) [34]. Education level was added to the model as an interaction term with the variable incidence of incontinence. Furthermore, as chronic illnesses are associated with life dissatisfaction [35], the total number of physical illnesses (e.g., diabetes) was added as a surrogate for somatic comorbidity as an independent variable (ranging from 0 to 11). As there is evidence that treatment of prostate cancer is one of the main causes of incontinence in men [5], we excluded individuals with cancer from the regression analysis in a further sensitivity analysis.

Data pertaining to the incidence of incontinence were collected as part of the face-to-face interview. Other variables (e.g. life satisfaction, number of physical diseases) were collected as part of the additional questionnaire completed individually.

### Statistical analysis

Panel regression models were used to examine the association between incontinence and life satisfaction longitudinally. Panel regression models have the advantage of being able to manage time-constant (unobserved) factors such as genetic disposition. These time-constant unobserved factors are treated as random variables in widely used regression models such as random effects (RE) regression models. However, when these unobserved time-constant factors are systematically correlated with the explanatory variables, these estimators will yield inconsistent estimates [36–38]. In contrast to these models, fixed effects (FE) regression models produce estimates which are consistent when this assumption is violated [36–38]. For this reason, FE regressions with cluster-robust standard errors [39] were used in the current study. The choice of our model is also motivated by Sargan Hansen tests which correspond to Hausman tests with cluster-robust standard errors. The finding of this test is that the FE estimator is consistent. For example, the Sargan-Hansen statistic was 186.59, *p* < .001 (main model, total sample), suggesting that there were systematic differences between the coefficients observed using the FE and the RE estimator.

Only changes within individuals over time are used in FE regressions (“Within-estimator”). Therefore, these changes are frequently interpreted in a causal sense (average treatment effect on the treated, ATET). However, the causal inference in our study is restricted because the treatment was not randomly assigned [38, 40].

Given the fact that only changes within individuals over time were used in the FE regressions, observable time-constant factors (e.g., education in older adults or sex) cannot be included as main effects in FE regressions. However, these time-constant factors can be used for group stratification or moderating factors (e.g., sex x incontinence). The potential gender differences were examined by including an interaction term in the main model. We also stratified the analysis by gender. Further details are given elsewhere [38, 40]. The statistical significance was defined as *p* value of .05 or smaller. Analyses were conducted using Stata 15.0 (StataCorp., College Station, Texas, USA).

## Results

### Sample characteristics

Table 1 displays the pooled (i.e., pooled across waves) descriptive characteristics stratified by sex of the observations used for the linear FE regressions with life satisfaction as outcome measure. Altogether 9869 observations (6959 individuals) were included in linear FE regression analysis. In total, 50.6% were male. The mean age in men was 64.9 years (±11.4 years), and the mean age in women was 62.7. In men, average life satisfaction score was 3.8 (±0.7) and in women average life satisfaction score was 3.8 (±0.8). Further details are displayed in Table 1.

**Table 1:** Characteristics of the observations included in linear Fixed-Effects Regressions (Waves 3–5, pooled, *n* = 9869)

	Men (*n* = 4995; 50.6%)	Women (*n* = 4874; 49.4%)	*p*-value
	N (%) / Mean (SD); Range	N (%) / Mean (SD); Range	
Age	64.9 (11.4); 40–95	62.7 (11.3); 40–93	<.001
Marital status: Divorced/Widowed/Single (Ref.: married, living together with spouse)	1060 (21.2%)	1675 (34.4%)	<.001
Employment status:			<.001
Employed	1680 (33.6%)	1686 (34.6%)	
Retired	2984 (59.8%)	2394 (49.1%)	
Other (not employed)	331 (6.6%)	794 (16.3%)	
Number of important people in regular contact	4.5 (2.8); 0–9	4.8 (2.8); 0–9	<.001
Education:			<.001
Low	161 (3.9%)	550 (13.6%)	
Medium	2085 (50.4%)	2238 (55.3%)	
High	1891 (45.7%)	1259 (31.1%)	
Self-rated health (from 1 = very good to 5 = bad)	2.5 (0.8); 1–5	2.5 (0.8); 1–5	.25
Physical functioning (from 0 = worst score to 100 = best score)	85.0 (21.5); 0–100	81.6 (23.6); 0–100	<.001
Absence of depression (CES-D < 18)	4785 (95.8%)	4500 (92.3%)	<.001
Absence of physician-diagnosed incontinence	4834 (96.8%)	4682 (96.1%)	.06
Life satisfaction	3.8 (0.7); 1–5	3.8 (0.8); 1–5	.15

Notes: *P*-values are based on Chi^2^-tests or t-tests, as appropriate. The Satisfaction with Life Scale (SWLS) was used to quantify life satisfaction [19]. The Center for Epidemiological Studies Depression Scale (CES-D) was used to quantify depression [32]. Physical functioning was measured by the subscale “Physical Functioning” of SF-36 Short Form Health Survey (0–100 range) [31]

It is worth noting that 152 individuals changed their status from ‘absence of incontinence’ to ‘incontinence’ over time. In other words: The number of physician-diagnosed incidental cases of incontinence in the study period was 152 (men: 64; women: 88).

### Regression analysis

In bivariate FE regression analysis, the onset of incontinence was associated with a decrease in life satisfaction in men (β = − 0.18, *p* < .05), but not in women (with significant gender differences, *p* < .05).

Results of multiple FE regression analysis are displayed in Table 2. FE regressions revealed that the onset of incontinence was associated with a decrease in life satisfaction in men (β = − 0.25, *p* < .01), but not in women. Gender differences (sex x incontinence) were significant (*p* < .05).

**Table 2:** Determinants of life satisfaction. Results of linear FE regression analysis

Independent variables	(1)	(2)	(3)	(4)
	Total sample	Men	Women	Total sample - with interaction term incontinence x sex
Age	0.02***	0.03***	0.01	0.02***
	(0.00)	(0.01)	(0.01)	(0.00)
Marital status: Divorced/Widowed/Single (Ref.: married, living together with spouse)	0.07	0.18*	−0.03	0.07
	(0.07)	(0.09)	(0.11)	(0.07)
Employment status: (Ref.: Employed)				
- Retired	−0.03	−0.04	− 0.02	− 0.03
	(0.05)	(0.07)	(0.07)	(0.05)
- Other (not employed)	−0.12**	−0.13+	− 0.13*	−0.12**
	(0.05)	(0.07)	(0.06)	(0.05)
Number of important people in regular contact	−0.00	−0.00	0.00	−0.00
	(0.00)	(0.00)	(0.00)	(0.00)
Self-rated health (from 1 = very good to 5 = bad)	−0.06***	−0.07**	− 0.04+	−0.06***
	(0.02)	(0.02)	(0.02)	(0.02)
Physical functioning (from 0 = worst score to 100 = best score)	0.00	0.00	0.00	0.00
	(0.00)	(0.00)	(0.00)	(0.00)
Depression (CES-D ≥ 18)	−0.21***	−0.22**	−0.21**	− 0.21***
	(0.05)	(0.08)	(0.06)	(0.05)
Presence of physician-diagnosed incontinence (Ref.: Absence of physician-diagnosed incontinence)	−0.08	− 0.25**	0.05	− 0.21*
	(0.06)	(0.09)	(0.08)	(0.09)
Interaction term: incontinence x sex (Ref.: men)				0.23*
				(0.11)
Constant	2.71***	1.98***	3.37***	2.71***
	(0.27)	(0.39)	(0.39)	(0.27)
Observations	9869	4995	4874	9869
Number of Individuals	6959	3539	3420	6959
R^2^	0.03	0.05	0.02	0.03

Notes: Beta-Coefficients are reported; Cluster-robust standard errors in parentheses. The Satisfaction with Life Scale (SWLS) was used to quantify life satisfaction [19]. The Center for Epidemiological Studies Depression Scale (CES-D) was used to quantify depression [32]. Physical functioning was measured by the subscale “Physical Functioning” of SF-36 (0–100 range) [31]. The Stata command for FE regression analysis (‘xtreg, fe’) include individuals with only one observation in calculating the number of observations because these individuals provide information about the variance components, the constant, the between R^2^ and so on. Nevertheless, it does not affect the beta-coefficients as well as the standard errors

*** *p* < .001, ** *p* < .01, * *p* < .05, + *p* < .10

With regard to control variables, a decrease in life satisfaction was associated with onset of depression in the total sample. Individuals who changed their employment status from employed to unemployed also exhibit lower scores of life satisfaction. Additionally, life satisfaction decreased with deterioration of self-rated health. Age was positively associated with life satisfaction. Please see Table 2 for further details (e.g., control variables).

In a sensitivity analysis, it was tested whether the impact of incontinence on life satisfaction differed by educational level. However, none of the interaction terms achieved statistical significance. In a further sensitivity analysis, the main model was extended by adding a sum score of physical illnesses. In a third sensitivity analysis, individuals with cancer were excluded (see Additional file 1). However, in terms of significance and effect size, the impact of incontinence on life satisfaction in men remained almost the same in all models.

## Discussion

### Main findings

Using a nationally representative sample of individuals aged 40 and above, the present longitudinal study examined the onset of incontinence on life satisfaction in the total sample and stratified by sex. Fixed effects regressions revealed that the onset of incontinence was associated with a decline in life satisfaction in men (β = −.25, *p* < .01), but not in the total sample and in women. Gender differences were significant (*p* < .05).

### Relation to previous research

Numerous studies have investigated the relation between incontinence and *HRQoL* [7, 8, 41]. However, it is worth emphasizing that this longitudinal study analyses the impact of incontinence on *life satisfaction*. Therefore, this section solely focuses on the relation between incontinence and life satisfaction. A few cross-sectional studies have shown that incontinence is associated with decreased satisfaction with life, mainly based on samples including both women and men [9–11]. Berg et al. [9] investigated life satisfaction (LSI-Z) in the oldest-old (80+). Based on the sample drawn from the Swedish OCTO-Twin study (*n* = 392) they showed UI is significantly related to decreased life satisfaction. In the mid-80s Herzog et al. [11] showed in a study based on probability sample of 747 women and 541 men aged 60 and older that urinary incontinence is related to low life satisfaction. Zhang and Yu [10] analyzed individuals from a clinical sample (geriatric out-patients; *n* = 200) and a randomly selected community sample (*n* = 150). Both samples were from the same area in Beijing. Individuals from the clinical sample with less frequent urinary incontinence reported significantly greater life satisfaction compared to individuals with more frequent symptoms of incontinence. It is worth mentioning that the samples differed significantly, e.g. male gender 63% vs. 36% in the clinical and in the community sample respectively (illiterate 14.5% vs. 46.7%).

While the aforementioned cross-sectional studies found an association between incontinence and life satisfaction, the onset of incontinence was not associated with life satisfaction in the total sample in our study. However, it is difficult to compare these cross-sectional findings with our results, given that our findings are based on nationally representative longitudinal data. These differences may be partially explained by self-selection. Self-selection is well recognized as a key challenge in non-experimental research. Self-selection means that individuals who score low in life satisfaction may have a higher probability of developing incontinence because they had adverse health outcomes prior to the onset of incontinence. FE-regressions, which were used in this study, are able to tackle bias related to self-selection by exploiting intra-individual changes over time.

In our longitudinal study, the onset of incontinence decreased satisfaction with life in men, but not in women, after adjusting for various potential confounders including mental and physical health. These differences may be explained by the fact that the onset of incontinence is a critical life event for men, but not for women. These expected differences in perceptions could be explained by the considerable gender differences in the prevalence of incontinence among older adults. Thus, while men may feel stigmatized when they experience incontinence, women may perceive incontinence rather as an aspect of the natural course of life. In addition, men experiencing incontinence may compare themselves with other men their age who are better off (i.e., do not have incontinence). These negative health comparisons may affect satisfaction with life in men [24]. In contrast, inter-individual comparisons generally did not affect life satisfaction in women.

Results remained virtually the same when individuals with cancer were excluded. Thus, we assume that the impact of incontinence on life satisfaction in men is not driven by consequences of prostate cancer treatment.

### Strengths and limitations

Some strengths are worth highlighting. For the present longitudinal study, data were drawn from a large, nationally representative study of community-dwelling individuals in the second half of life. Whether the onset of incontinence was associated with a change in life satisfaction over the course of 6 years was examined. Life satisfaction was measured using a well-established scale. Unobserved heterogeneity, a main challenge in large survey studies and life satisfaction research, was addressed by using FE regressions in the current study. Unlike previous studies based on self-reported incontinence, physician-diagnosed presence of incontinence was reported by the individuals in this study.

On the other hand, some limitations are worth noting. A small sample selection bias have been observed in the DEAS study. Though quite small, panel attrition was observed in this study. Nevertheless, intense efforts have been made to decrease attrition, and the retention rate has increased. Please see Klaus et al. [27] for further details. The distribution of sociodemographic factors in the sample is very close to that of the German population [42]. Due to the sensitive question concerning incontinence, bias may have occurred. The physician-diagnosed presence of incontinence was quantified in the DEAS study without distinguishing between UI and FI. However, only approximately 6.0% of women and 1.9% of men reported both UI and FI [4]. Furthermore, the prevalence of FI, without symptoms for both FI and UI, is estimated to be about 8%, for both community-dwelling women and men [4]. Nevertheless, further research is required that considers the severity of urinary and fecal incontinence. Moreover, other time-varying factors (e.g., cognitive decline, walking speed) may affect our relationship of interest. Future research is required to examine these factors.

## Conclusions

Incontinence affects life satisfaction among men aged 40 and over. Surprisingly, the onset of incontinence was not associated with a decline in life satisfaction in women.

In general, individuals markedly underreport the presence of incontinence and do not discuss it with their general practitioners. These conversations may be beneficial in order to discuss the treatment options. These interventional strategies to mitigate or postpone incontinence might contribute to maintaining life satisfaction in men. Future research is required to examine this relationship in further detail. For example, coping strategies (e.g., self-efficacy) in this relationship could be examined.

### Supplementary information

**Additional file 1.** Determinants of life satisfaction. Results of linear FE regression analysis (without individuals with cancer).

## Data Availability

The data used in this study are third-party data. The anonymized data sets of the DEAS (1996, 2002, 2008, 2011, and 2014) are available for secondary analysis. The data has been made available to scientists at universities and research institutes exclusively for scientific purposes. The use of data is subject to written data protection agreements. Microdata of the German Ageing Survey (DEAS) is available free of charge to scientific researchers for non-profitable purposes. The Research Data Centre of the German Centre of Gerontology provides access and support to scholars interested in using DEAS for their research. However, for reasons of data protection, signing a data distribution contract is required before data can be obtained. Please see for further Information (data distribution contract): https://www.dza.de/en/fdz/german-ageing-survey/access-to-deas-data.html

## Appendix

### List of illnesses

A: High cholesterol

B: Diabetes, high blood sugar levels

C: High blood pressure

D: Heart attack, angina pectoris

E: Cardiac insufficiency including coronary artery disease

F: Stroke

G: Circulatory disorders in the brain

H: Circulatory disorders in the legs

J: Joint degeneration (arthrosis) of the hips, knees, or spine

K: Osteoporosis

L: Inflammatory joint or spinal disease (arthritis or rheumatoid arthritis)

M: Chronic pulmonary disease (e.g., chronic bronchitis, pulmonary emphysema)

N: Cancer, malignant tumor (including leukemia)

O: Stomach ulcer, intestinal ulcer

P: Incontinence

Q: Mental illness (e.g. panic attacks, depression, psychosis)

R: Parkinson’s disease

S: Glaucoma or macular degeneration

T: Other chronic disease or health condition (only longer-term or recurring diseases) please specify:

## Abbreviations

ATET: Average treatment effect on the treated
CES-D: Center for Epidemiological Studies Depression Scale
DEAS: German Ageing Survey
FE: Fixed effects
FI: Fecal incontinence
HRQoL: Health-related quality of life
ICS: International Continence Society
ISCED-97: International Standard Classification of Education
RE: Random effects
SWB: Subjective well-being
SWLS: Satisfaction with Life Scale
UI: Urinary incontinence

## References

[1] Abrams P, Cardozo L, Fall M, Griffiths D, Rosier P, Ulmsten U (2002). The standardisation of terminology of lower urinary tract function: report from the standardisation sub-committee of the international continence society. Neurourol Urodyn.

[2] Norton C, Whitehead W, Bliss D, Metsola P, Tries J, Abrams A, Cardozo L, Khoury S, Wein A (2005). Conservative treatment and pharmacological management of faecal incontinence in adults. (2005) Incontinence: Volume 2-Management.

[3] Buckley BS, Lapitan MC (2010). Prevalence of urinary incontinence in men, women, and children--current evidence: findings of the fourth international consultation on incontinence. Urology..

[4] Wu JM, Matthews CA, Vaughan CP, Markland AD (2015). Urinary, fecal, and dual incontinence in older U.S. Adults. J Am Geriatr Soc.

[5] Moore KN, Gray M (2004). Urinary incontinence in men: current status and future directions. Nurs Res.

[6] Markland AD, Goode PS, Redden DT, Borrud LG, Burgio KL (2010). Prevalence of urinary incontinence in men: results from the national health and nutrition examination survey. J Urol.

[7] Tikkinen KA, Agarwal A, Griebling TL (2013). Epidemiology of male urinary incontinence. Curr Opin Urol.

[8] Malmsten UG, Molander U, Peeker R, Irwin DE, Milsom I (2010). Urinary incontinence, overactive bladder, and other lower urinary tract symptoms: a longitudinal population-based survey in men aged 45-103 years. Eur Urol.

[9] Berg AI, Hassing LB, Nilsson SE, Johansson B (2009). “As long as I’m in good health”. The relationship between medical diagnoses and life satisfaction in the oldest-old. Aging Clin Exp Res.

[10] Zhang AY, Yu LC (1998). Life satisfaction among Chinese elderly in Beijing. J Cross Cult Gerontol.

[11] Herzog AR, Fultz NH, Brock BM, Brown MB, Diokno AC (1988). Urinary incontinence and psychological distress among older adults. Psychol Aging.

[12] Dugan E, Cohen SJ, Robinson D, Anderson R, Preisser J, Suggs P (1998). The quality of life of older adults with urinary incontinence: determining generic and condition-specific predictors. Qual Life Res.

[13] Chang PL, Tsai LH, Huang ST, Wang TM, Hsieh ML, Tsui KH (1998). The early effect of pelvic floor muscle exercise after transurethral prostatectomy. J Urol.

[14] Edwards DF, Hahn M, Dromerick A (2006). Post stroke urinary loss, incontinence and life satisfaction: when does post-stroke urinary loss become incontinence?. Neurourol Urodyn.

[15] Enkvist A, Ekstrom H, Elmstahl S (2012). Life satisfaction (LS) and symptoms among the oldest-old: results from the longitudinal population study called good aging in Skane (GAS). Arch Gerontol Geriatr.

[16] Pinto JM, Neri AL (2013). Factors associated with low life life satisfaction in community-dwelling elderly: FIBRA study. Cad Saude Publica.

[17] Torrance GW (1987). Utility approach to measuring health-related quality of life. J Chronic Dis.

[18] Karimi M, Brazier J (2016). Health, health-related quality of life, and quality of life: what is the difference?. Pharmaco Economics..

[19] Diener E, Emmons RA, Larsen RJ, Griffin S (1985). The satisfaction with life scale. J Pers Assess.

[20] Diener E, Pressman SD, Hunter J, Delgadillo-Chase D (2017). If, why, and when subjective well-being influences health, and future needed research. Appl Psychol Health Well Being.

[21] Diener E (1984). Subjective well-being. Psychol Bull.

[22] Diener E, Chan MY (2011). Happy people live longer: subjective well-being contributes to health and longevity. Appl Psychol.

[23] Goode PS, Burgio KL, Richter HE, Markland AD (2010). Incontinence in older women. JAMA.

[24] Hajek A, Konig HH (2016). Negative health comparisons decrease affective and cognitive well-being in older adults. Evidence from a Population-Based Longitudinal Study in Germany Frontiers in psychology. Front Psychol.

[25] Elstad EA, Taubenberger SP, Botelho EM, Tennstedt SL (2010). Beyond incontinence: the stigma of other urinary symptoms. J Adv Nurs.

[26] Clark AE, Georgellis Y (2013). Back to baseline in Britain: adaptation in the British household panel survey. Economica..

[27] Klaus D, Engstler H, Mahne K, Wolff JK, Simonson J, Wurm S (2017). Cohort Profile: The German Ageing Survey (DEAS). Int J Epidemiol.

[28] Neller K (2005). Kooperation und Verweigerung. Eine non-response-Studie [co-operation and refusal: a non-response study]. ZUMA Nachrichten.

[29] Pavot W, Diener E (1993). Review of the satisfaction with life scale. Psychol Assess.

[30] Charlson M, Szatrowski TP, Peterson J, Gold J (1994). Validation of a combined comorbidity index. J Clin Epidemiol.

[31] Ware JE, Sherbourne CD (1992). The MOS 36-item short-form health survey (SF-36): I. conceptual framework and item selection. Med Care.

[32] Radloff LS (1977). The CES-D scale a self-report depression scale for research in the general population. Appl Psychol Meas.

[33] Fayers PM, Sprangers MA (2002). Understanding self-rated health. Lancet.

[34] UNESCO (2006). International Standard Classification of Education. ISCED 1997. Re-edition ed.

[35] Strine TW, Chapman DP, Balluz LS, Moriarty DG, Mokdad AH (2008). The associations between life satisfaction and health-related quality of life, chronic illness, and health behaviors among U.S. community-dwelling adults. J Community Health.

[36] Cameron AC, Trivedi PK (2005). Microeconometrics: methods and applications.

[37] Greene WH (2012). Econometric analysis.

[38] Wooldridge JM (2010). Econometric analysis of cross section and panel data.

[39] Stock JH, Watson MW (2008). Heteroskedasticity-robust standard errors for fixed effects panel data regression. Econometrica..

[40] Brüderl J, Ludwig V, Wolf C (2015). Fixed-effects panel regression. The Sage handbook of regression analysis and causal inference.

[41] Abrams P, Smith AP, Cotterill N (2015). The impact of urinary incontinence on health-related quality of life (HRQoL) in a real-world population of women aged 45-60 years: results from a survey in France, Germany, the UK and the USA. BJU Int.

[42] Klaus D, Engstler H, Mahne K, Wolff JK, Simonson J, Tesch-Römer C (2017). Data and methods of German Ageing Survey [Daten und Methoden des Deutschen Alterssurveys]. Ageing in Social Change Two Decades of the German Ageing Survey [Altern im Wandel: Zwei Jahrzehnte Deutscher Alterssurvey (DEAS)].

