# Supplementary material for: How does the onset of incontinence affect satisfaction with life among older women and men? Findings from a nationally representative longitudinal study (German Ageing Survey)
Source: Health Qual Life Outcomes. 2020 Jan 28;18:16. doi: 10.1186/s12955-020-1274-y (PMC6985999; doi:10.1186/s12955-020-1274-y)
Supplement: Supplementary file 1 — Additional file 1. Determinants of life satisfaction. Results of linear FE regression analysis (without individuals with cancer). [file 12955_2020_1274_MOESM1_ESM.docx]

Additional file 1

Table S1. Determinants of life satisfaction. Results of linear FE regression analysis (without individuals with cancer)

|  | (1) | (2) | (3) | (4) |
| --- | --- | --- | --- | --- |
| Independent variables | Total sample | Men | Women | Total sample - with interaction term incontinence x sex |
|  |  |  |  |  |
| Age | 0.02*** | 0.03*** | 0.01 | 0.02*** |
|  | (0.00) | (0.01) | (0.01) | (0.00) |
| Marital status: Divorced/Widowed/Single (Ref.: married, living together with spouse) | 0.04 | 0.15 | -0.05 | 0.04 |
|  | (0.08) | (0.10) | (0.11) | (0.08) |
| Employment status: Retired (Ref.: Employed) | -0.05 | -0.06 | -0.03 | -0.05 |
|  | (0.05) | (0.07) | (0.07) | (0.05) |
| Other (not employed) | -0.12** | -0.13+ | -0.12* | -0.12** |
|  | (0.05) | (0.07) | (0.06) | (0.05) |
| Number of important people in regular contact | -0.00 | -0.00 | -0.00 | -0.00 |
|  | (0.00) | (0.00) | (0.01) | (0.00) |
| Self-rated health (from 1 = very good to 5 = bad) | -0.06** | -0.06** | -0.05+ | -0.06** |
|  | (0.02) | (0.02) | (0.02) | (0.02) |
| Physical functioning (from 0 = worst score to 100 = best score) | 0.00 | 0.00 | 0.00 | 0.00 |
|  | (0.00) | (0.00) | (0.00) | (0.00) |
| Depression (CES-D ≥ 18) | -0.19*** | -0.24** | -0.17** | -0.20*** |
|  | (0.05) | (0.07) | (0.06) | (0.05) |
| Presence of physician-diagnosed incontinence (Ref.: Absence of physician-diagnosed incontinence) | -0.10 | -0.26** | 0.01 | -0.21* |
|  | (0.06) | (0.09) | (0.08) | (0.09) |
| Interaction term: incontinence x sex (Ref.: men) |  |  |  | 0.19 |
|  |  |  |  | (0.12) |
| Constant | 2.77*** | 2.05*** | 3.42*** | 2.77*** |
|  | (0.28) | (0.41) | (0.40) | (0.28) |
|  |  |  |  |  |
| Observations | 9,130 | 4,587 | 4,543 | 9,130 |
| Number of Individuals | 6,540 | 3,298 | 3,242 | 6,540 |
| R² | 0.03 | 0.04 | 0.02 | 0.03 |

Notes: Beta-Coefficients are reported; Cluster-robust standard errors in parentheses. The Satisfaction with Life Scale (SWLS) was used to quantify life satisfaction (13). The Center for Epidemiological Studies Depression Scale (CES-D) was used to quantify depression (24). Physical functioning was measured by the subscale “Physical Functioning” of SF-36 Short Form Health Survey (0-100 range) (23). It is worth noting that the Stata command for FE regression analysis (‘xtreg, fe’) include individuals with only one observation in calculating the number of observations because these individuals provide information about the variance components, the constant, the between R² and so on. Nevertheless, it does not affect the beta-coefficients as well as the standard errors. *** p<0.001, ** p<0.01, * p<0.05, + p<0.10.
